# Risk of transient vision loss after intravitreal aflibercept using vial-prepared vs. the novel prefilled syringe formulation

**DOI:** 10.3389/fmed.2023.1295633

**Published:** 2023-10-25

**Authors:** Julian E. Klaas, Vinh Bui, Niklas Maierhofer, Benedikt Schworm, Mathias Maier, Siegfried G. Priglinger, Jakob Siedlecki

**Affiliations:** ^1^Department of Ophthalmology, Ludwig-Maximilians-University, Munich, Germany; ^2^Department of Ophthalmology, Technical University, Munich, Germany

**Keywords:** Eylea, aflibercept, prefilled syringe, choroidal neovascularization, age related macular degeneration, diabetic macular edema, retinal vein occlusion

## Abstract

**Purpose:**

To compare the risk of transient vision loss (TVL) probably attributable to a severe intraocular pressure spike after intravitreal aflibercept application using the novel prefilled syringe (PFS) vs. the established vial system (VS).

**Methods:**

Datasets of the intravitreal injection service of the Ludwig Maximilians-University Munich and the Technical University Munich, Germany, were screened for documentation of TVL after intravitreal injection of aflibercept. The observation period included two full months prior to the introduction of the novel PFS and two months afterwards. TVL was defined as loss of perception of hand motion for a duration of >30 s.

**Results:**

Over a period of four months, 1720 intravitreal injections of aflibercept were administered in 672 patients. There were 842 injections with the old VS, and 878 injections using the novel PFS. Using the VS, TVL was noted during two injections (0.24%) in two patients, as compared to 11 cases of TVL (1.25%) in 10 patients with the PFS (*p* = 0.015). Using the PFS, patients had a 5.3-fold risk of TVL as compared to the VS (OR: 5.33; 95% CI: 1.2–24.1; *p* = 0.0298).

**Conclusion:**

There was a more than five-fold risk of TVL using the novel pre-filled aflibercept syringe as compared to the established vial system. During informed consent, this risk should be discussed.

## 1. Introduction

In the last decade, the introduction of intravitreal vascular endothelial growth factor (VEGF) inhibitors has revolutionized the treatment of a multitude of retinal diseases (1). Currently, ranibizumab, aflibercept and brolucizumab possess approval for the treatment of one or more mostly macular disorders, while bevacizumab is being widely used as an off-label alternative (1). In this competitive field, pharmaceutical innovation, e.g., the modification of posology, tissue penetration or sheer efficacy in retinal drying is strategically addressed to secure and expand market share.

In this context, aflibercept was approved and launched as a novel prefilled syringe (PFS) in the USA in August 2019 and in the European Union in April 2020 in order to potentially enable a more streamlined and efficient injection procedure (2, 3). Shortly after introduction, surgeons at the Department of Ophthalmology of both Technical University Munich (TUM) and Ludwig Maximilians-University (LMU) experienced an unusually high incidence of transient vision loss (TVL) due to suspected intraocular pressure spikes directly after the intravitreal application of aflibercept using the novel PFS. Due to the loss of visual acuity suggesting transient central retinal artery occlusion (4), associated long-term damage, e.g., permanent arterial occlusion (5), cannot be excluded. Apart from a recent publication by Gallagher et al. reporting the first case series of transient retinal artery occlusion associated with the aflibercept PFS in 5 eyes of 4 patients (6), we are not aware of any clinical studies that have investigated this issue. Therefore, this retrospective multicenter cross-sectional study was designed to compare the incidence of TVL using the old vial-based injection system vs. the novel PFS system.

## 2. Materials and methods

In LMU, the novel aflibercept PFS (Bayer, Leverkusen, Germany) was introduced on July 27th, 2020; in TUM, the introduction date was June 1st, 2020. To determine the incidence of TVL, two months (9 calendar weeks) prior to and after switching from the vial to the novel PFS system were defined as observation period. Thus, the observation period at LMU was May 25th until July 26th 2020 for the vial system, and July 27th until September 27th 2020 for the novel PFS. At TUM, the respective observation periods were March 30th until May 31st 2020 for the vial system, and June 1st until August 2nd 2020 for the novel PFS system.

To assess the incidence of TVL, anonymized datasets generated from both intravitreal injection service databases covering the aforementioned observation period were screened for a documentation of TVL associated with an aflibercept injection as defined below as well as required pharmacologic or surgical interventions (e.g., application of eye-pressure lowering agents or paracentesis). Because the research was performed on anonymized data, no prior approval of the respective ethics committees was required.

### 2.1. Injection procedure

All patients were injected in designated intravitreal injection operating rooms according to established hygiene protocols and standard operating procedures. Prior to entering the operating room, all patients were dressed in a hygienic overcoat and equipped with a surgical cap. After topical anesthesia, eyes, lids and periocular skin were disinfected with 1% povidone iodine in supine position. The eyes were then draped and a lid speculum was inserted. Aflibercept was injected in a distance of 3.5 to 4.0 mm posteriorly to the limbus using either Becton Dickinson syringes filled from the aflibercept vial or the novel PFS. A 30 Gauge needle was used in all cases. The correct injection volume of 50 μL was prepared according to the package insert by one of two specialized nurses and checked by the surgeon prior to injection (LMU), or both prepared and checked by the surgeon (TUM). Hereby, the base of the plunger dome (not the tip of the dome) was aligned with the dosing mark. Where possible, care was taken to ensure that the plunger was not aligned proximal or distal to the mark. Directly after injection, visual acuity of hand motion and above was examined at a distance of 1 foot. No changes in equipment or injection technique occurred during the observation period.

### 2.2. Definition of TVL

TVL was defined as loss of hand motion immediately or within the first minute after the injection for a duration of >30 s with a hard eye ball on palpation. The need for paracentesis was assessed individually by the surgeon based on the duration of loss of hand motion, palpation of the globe and the individual spontaneous tendency for resolution including the patient’s subjective course of symptoms within the first **2** min. Epidemiological data was obtained from the anonymized datasets to correlate TVL during aflibercept therapy with age, gender, ocular comorbidities as well as history of prior TVL during intravitreal injection and the numbers of aflibercept applications before the date of TVL.

### 2.3. Statistical analysis

All data were gathered and analyzed in Microsoft Excel spreadsheets (Version 16.23 for Mac; Microsoft, Redmond, WA, United States). Statistical analysis was performed in SPSS Statistics 26 (IBM Germany GmbH, Ehningen, Germany). The level to indicate statistical significance was defined as *p* < 0.05. Statistical analyses of intra-group differences were performed using a Chi-Square test and Fisher’s exact test. Graphs and diagrams were plotted in Microsoft Excel.

## 3. Results

**Figure 1:**
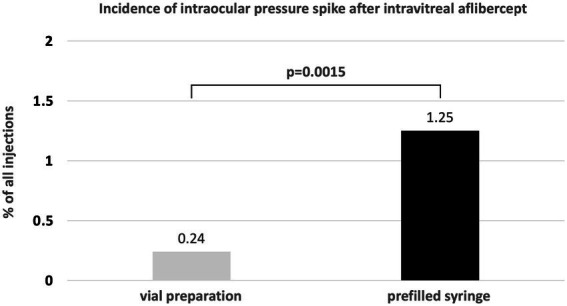
Incidence of transient vision loss due to ocular hypertension with the aflibercept vial system vs. the novel PFS system.

In total, 1720 injection procedures of intravitreal aflibercept were performed during the defined time span in 672 patients. Of these, 842 (49.0%) were performed with the old vial system, and 878 (51.0%) were performed with the new PFS. With the vial system, 2 eyes of 2 patients (0.24%) experienced TVL; with the new PFS, 11 eyes (1.25%) of 10 patients experienced TVL (*p* = 0.015; Figure **1**). Six different surgeons were involved in all cases of TVL (2 with the vial system, 5 with the PFS).

The two patients experiencing TVL with the vial system were both men (100%) with a mean age of 83 ± 1.4 years. The reason for treatment was neovascular AMD in both eyes (100%). There was one right and one left eye, and there were 32 and 1 previous aflibercept injections prior to TVL. None of these eyes (0%) had experienced TVL prior to this study. One eye (50%) had a history of ocular hypertension with IOP values around 25 mmHg. These two events of TVL occurred to two different surgeons. None of these eyes required paracentesis.

The 10 patients experiencing TVL with the PFS were equally male/female (50/50%) with a mean age of 75.6 ± 9.2 years. The reason for treatment in the 11 eyes was neovascular AMD in 6 eyes (54.5%), diabetic macular edema in 2 eyes (18.2%), macular edema due to central retinal vein occlusion in 2 eyes (18.2%) and branch retinal vein occlusion in 1 eye (9.1%). There were 3 right (27.3%) and 8 left eyes (72.7%), and there were mean 15.1 ± 14.7 previous aflibercept injections prior to TVL. One of these eyes (9.1%) had experienced one event of TVL prior to this study. Five eyes (45.5%) had a history of manifest glaucoma. The 11 events of TVL with the aflibercept PFS occurred in 5 surgeons (surgeon 1: 4 events; surgeon 2: 3 events; surgeon 3: 2 events; surgeons 4 and 5: 1 event). Eight out of the 11 cases of TVL (72.7%) required a paracentesis, which was performed at least once by 5 out of the 6 surgeons (83.3%). Table 1 summarizes the demographic and clinical characteristics of all patients experiencing TVL before and after the introduction of PFS.

**Table 1:** Characteristics of patients with transient vision loss (TVL) after intravitreal administration of aflibercept, using the established vial system vs. the newer PFS system.

Characteristics of patients with TVL	Vial system	Prefilled syringe
Number of eyes with TVL/total, *no. (%)*	2 / 842 (0.24%)^**π**^	11 / 878 (1.25%)^**ƒ**^
Sex		
Male, *no. (%)*	2 (100)	5 (50)
Female, *no. (%)*	0 (0)	5 (50)
Age *mean ± SD, in years*	83 ± 1.4	75.6 ± 9.2
Eye		
Right, *no. (%)*	1 (50)	3 (27.3)
Left, *no. (%)*	1 (50)	8 (72.7)
Prior injections, *mean no. ± SD*	16.5 ± 15.5	15.1 ± 14.7
Reason for treatment*		
Neovascular AMD, *no. (%)*	2 (100)	6 (54.5)
Diabetic macular edema, *no. (%)*	0 (0)	2 (18.2)
Central retinal vein occlusion, *no. (%)*	0 (0)	2 (18.2)
Branch retinal vein occlusion, *no. (%)*	0 (0)	1 (9.1)
Other, *no. (%)*	0 (0)	0 (0)
Other ocular comorbidities*		
Ocular hypertension, *no. (%)*	1 (50)	0 (0)
Glaucoma, *no. (%)*	0 (0)	5 (45.5)
Eyes with TVL prior to observation period, no. (%)*	0 (0)	1 (9.1)°
Surgeons involved in TVL events, *no.*	2	5^
Need for paracentesis, *no.* ^¥^*	0 (0)	8 (72.7) ^Ω^

1 ^π^2 eyes with TVL in 2 patients.

ƒ 11 eyes with TVL in 10 patients.

* percentage of the total number of eyes with TVL.

° one prior TVL event was documented in this case.

^ surgeon 1: 4 events; surgeon 2: 3 events; surgeon 3: 2 events; surgeons 4 and 5: 1 event.

¥ the need for paracentesis was assessed individually by the surgeon based on the duration of loss of perception of hand motion, palpation of the globe and the individual spontaneous tendency for resolution including the patient’s subjective course of symptoms within the first 2 min.

Ω a paracentesis was performed at least once by 5 out of the 6 surgeons (83.3%).

**Figure 2:**
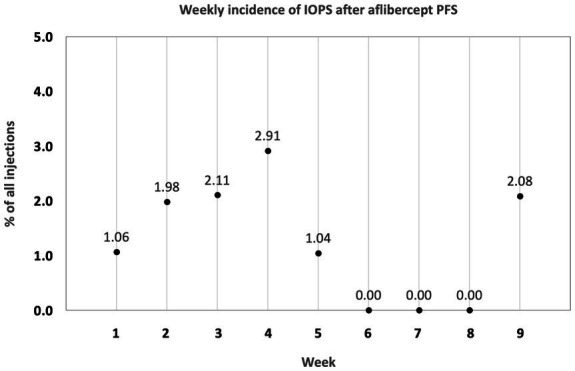
Distribution of transient vision loss (TVL) due to an intraocular pressure spike (IOPS) after aflibercept PFS introduction. TVL was consistently seen from week one through week 9.

Overall, the risk of TVL was 5.3 times higher with the PFS as compared to the old vial system (OR: 5.33; 95% CI: 1.2–24.1; *p* = 0.0298). Using the aflibercept PFS, events of TVL were homogenously distributed over the 9 week observation period with an incidence of 1.06% in the first, 1.98% in the second, 2.11% in the third, 2.91% in the fourth, 1.04% in the fifth, 0% in the sixth to eighth and 2.08% in the ninth week (Figure 2). No other serious adverse events (e.g., endophthalmitis, retinal detachment, hemorrhage) were observed during the study period.

## 4. Discussion

This study suggests a higher incidence of intraocular pressure spikes with transient vision loss immediately after intravitreal injection of aflibercept when using the novel PFS formulation as compared to the established vial system with conventional 1.0 mL insulin syringes. During a time span of 4 months with 1720 injections in 672 patients, significant spikes impacting visual acuity directly after the procedure were seen in 0.24% with the vial system, and in 1.25% with the novel PFS, resulting in an odds ratio of 5.33 (95% CI: 1.2–24.1; *p* = 0.0298).

As spikes were not defined by absolute measurements of intraocular pressure (e.g., with Goldmann applanation), but rather defined as transient vision loss with a palpatory hard eye ball, occlusion of the central retinal artery has to be assumed to be the driving pathomechanism (4). Since subsequent permanent retinal artery occlusion cannot be excluded (5) and optic nerve heads at risk (e.g., glaucoma) could be substantially harmed by such spikes (e.g., wipeout) (7), the complication reported herein represents a significant safety issue, even if rapid treatment can usually avoid long-term damage.

Concerning treatment of the cases reported herein, the standard operating procedures of both clinics suggest paracentesis with drainage of aqueous humor in eyes that do not regain visual acuity within 30–60 s. Interestingly, the majority (73%) of eyes treated with the PFS required urgent paracentesis to relieve the pressure since no spontaneous amelioration of intraocular perfusion was observed. In addition to the risk of the pressure spike itself, the majority of eyes complicated by such spikes will also undoubtedly be exposed to secondary complications, e.g., endophthalmitis, hemorrhage or hypotony in the case of leakage through the paracentesis, even if rare. We therefore believe that, especially in a procedure performed so frequently each day all over the world, these safety concerns should be taken seriously.

In the present study, the incidence of TVL found in eyes treated with the new PFS was higher not only compared to the vial system, but also compared to the known incidence of acute vision loss after intravitreal injection, which has been reported to be 0.14% (8).Two main hypotheses could explain why spikes in intraocular pressure might exacerbate with the novel PFS as compared to the vial system or compared to the ranibizumab prefilled syringe (Novartis, Basel, Switzerland). Recently, Gallagher et al. reported the first case series of transient retinal artery occlusion with the aflibercept PFS in 5 eyes of 4 patients (6). In this article, the authors refer to the different diameters of the conventional 1 mL syringe as compared to the aflibercept PFS, resulting in different calculated internal surface areas of 32 mm^2^ for the aflibercept PFS, and 16.6 mm^2^ for the 1 mL syringe (6). In fact, the authors were able to show that six injectors performing 20 sham injections each (120 sham injections in total) accidentally sham-injected a volume of 0.07 mL or more in 21% of procedures with the PFS. Thus, even slight errors in plunger alignment can lead to much higher variability in injection volume in the aflibercept PFS (6). This was confirmed by a more recent *in vitro* study, which evaluated the accuracy of injection volumes by 20 ophthalmologists using the PFS vs. a 1 mL BD syringe and a micro scale (9). The authors found that both injection volume and variability were significantly higher with the PFS system than with the previously used system (9). This phenomenon seems to be reproducible when compared to any syringe with a smaller diameter, including other prefilled formulations such as ranibizumab, as was recently shown by Hinkle et al. (10). However, we believe that even with a correctly dosed PFS, sharp rises of intraocular pressure could be provoked by different injection speeds and volume jets due to the higher diameter of the novel PFS resulting in both a shorter distance that the plunger needs to be pushed (aflibercept PFS 2.2 mm; 1 mL syringe: 4.2 mm) and the perception of a higher initial resistance (6). This assumption is supported by previous findings that higher IOP spikes after intravitreal injections have been documented in association with a smaller needle bore size (11). The same should be true for a constant needle bore size (30 gauge) but wider syringe diameters: assuming similar plunger speed applied by the surgeon, the flow rate of aflibercept into the vitreous cavity in an almost doubled surface area should be proportionally higher.

At the recent Pharmacovigilance Risk Assessment Committee (PRAC) meeting held on March 8th 2021, the European Medicines Agency (EMA) issued a statement that a “higher-than-expected proportion of cases of increased pressure inside the eye (intraocular pressure) were reported with the Eylea pre-filled syringes” (12), strengthening the importance of the matter. As “incorrect handling” was given as most probable cause of the problem, the acuity of the problem should be communicated to the ophthalmological community and additional training should be provided. In our study, however, spikes in IOP occurred to six different surgeons who are accustomed to double-checking the correct injection volume. Moreover, these spikes did not occur only shortly after the PFS introduction, but were consistently observed over the course of the 9 weeks evaluated, starting with 1% at week 1, 3% at week 4, and 2% at week 9. All adverse events reported in this study were communicated to the manufacturer and the relevant regulatory authorities.

Limitations of our study include its retrospective nature and the lack of IOP measurements in the cases reported. Since these spikes with central artery occlusion represent a severe acute complication, these patients were kept on the operating table for a possible immediate intervention and were not transferred to a slit lamp for Goldmann applanation tonometry. Furthermore, due to the low absolute number of TVL events, the confidence intervals are rather wide, indicating that better statistical power could be achieved with an even larger sample size, which may be difficult however, due to the low incidence of TVL, especially in the „control group“, and may require a multi-centric study with a high case number.

In conclusion, our analysis of a large database of 1720 intravitreal aflibercept procedures suggests that the current aflibercept PFS has a more than fivefold risk of significant transient intraocular pressure spikes over the conventional vial system used with a 1 mL syringe. Since these spikes were accompanied by transient vision loss, central retinal artery occlusion has to be assumed, making this a severe complication. As PFS systems represent an easier-to-use and potentially more efficient way to administer intravitreal substances with a lower intraocular infection risk (13), the current aflibercept PFS system may benefit from a redesign to make it less vulnerable to the complication described herein (13).

## Data availability statement

The raw data supporting the conclusions of this article will be made available by the authors, upon reasonable request.

## Author contributions

JK: Conceptualization, Data curation, Investigation, Writing – original draft, Writing – review & editing. VB: Data curation, Investigation, Methodology, Validation, Writing – review & editing. NM: Data curation, Investigation, Methodology, Writing – review & editing. BS: Conceptualization, Investigation, Methodology, Writing – review & editing. MM: Supervision, Writing – review & editing. SP: Data curation, Project administration, Supervision, Writing – review & editing. JS: Conceptualization, Data curation, Methodology, Supervision, Writing – original draft.
